# The Algicidal Bacterium *Kordia algicida* Shapes a Natural Plankton Community

**DOI:** 10.1128/AEM.02779-18

**Published:** 2019-03-22

**Authors:** Arite Bigalke, Nils Meyer, Lydia Alkistis Papanikolopoulou, Karen Helen Wiltshire, Georg Pohnert

**Affiliations:** a Institute for Inorganic and Analytical Chemistry, Bioorganic Analytics, Friedrich Schiller University Jena, Jena, Germany; b Alfred Wegener Institut, Helmholtz-Zentrum für Polar- und Meeresforschung, Biologische Anstalt Helgoland, Bremerhaven, Germany; c Alfred Wegener Institut, Helmholtz-Zentrum für Polar- und Meeresforschung, Biologische Anstalt Helgoland, Wadden Sea Station, List, Germany; d Max Planck Institute for Chemical Ecology, Jena, Germany; Chinese Academy of Sciences

**Keywords:** Helgoland Roads, North Sea, phytoplankton, algicidal bacteria, community interaction, microbial loop, plankton succession

## Abstract

Plankton communities consist of complex microbial consortia that change over time. These fluctuations can be only partially explained by limiting resources. Biotic factors such as herbivores and pathogens also contribute to the control of algal blooms. Here we address the effects of algicidal bacteria on a natural plankton community in an indoor enclosure experiment. The algicidal bacteria, introduced into plankton taken directly from the North Sea during a diatom bloom, caused the rapid decline of the bloom-forming *Chaetoceros socialis* within only 1 day. The haptophyte *Phaeocystis*, in contrast, is resistant to the lytic bacteria and could benefit from the removal of the competitor, as indicated by an onset of a bloom in the treated enclosures. This cascading effect caused by the bacterial pathogen accelerated the succession of *Phaeocystis*, which bloomed with a delay of only several weeks in the *in situ* waters at Helgoland Roads in the North Sea. The algicidal bacteria can thus modulate the community within the limits of the abiotic and biotic conditions of the local environment. Implications of our findings for plankton ecosystem functioning are discussed.

**IMPORTANCE** Plankton communities change on a seasonal basis in temperate systems, with distinct succession patterns; this is mainly due to algal species that have their optimal timing relative to environmental conditions. We know that bacterial populations are also instrumental in the decay and termination of phytoplankton blooms. Here, we describe algicidal bacteria as modulators of this important species succession. Upon treatment of a natural plankton consortium with an algicidal bacterium, we observed a strong shift in the phytoplankton community structure, compared to controls, resulting in formation of a succeeding *Phaeocystis* bloom. Blooms of this alga have a substantial impact on global biogeochemical and ecological cycles, as they are responsible for a substantial proportion of primary production during spring in the North Sea. We propose that one of the key factors influencing such community shifts may be algicidal bacteria.

## Abstract (precis)

Plankton communities change on a seasonal basis in temperate systems, with distinct succession patterns; this is mainly due to algal species that have their optimal timing relative to environmental conditions. We know that bacterial populations are also instrumental in the decay and termination of phytoplankton blooms. Here, we describe algicidal bacteria as modulators of this important species succession. Upon treatment of a natural plankton consortium with an algicidal bacterium, we observed a strong shift in the phytoplankton community structure, compared to controls, resulting in formation of a succeeding *Phaeocystis* bloom. Blooms of this alga have a substantial impact on global biogeochemical and ecological cycles, as they are responsible for a substantial proportion of primary production during spring in the North Sea. We propose that one of the key factors influencing such community shifts may be algicidal bacteria.

## INTRODUCTION

Phytoplankton successions can, to a large extent, be explained by limiting resources available to the respective primary producers. Changing nutrient availability, light, and temperature provide fluctuating optima for specific members of the plankton community and lead to the observed complex community dynamics over time (1–4). Additionally, biotic interactions shape the community. These include (selective) predation by herbivores, allelopathic interactions between the primary producers, and control of algal development and decay by bacteria (5). Tight coupling of bacteria and phytoplankton leads to recurring patterns of microbial clades during phytoplankton spring blooms, which are correlated with deterministic principles, such as substrate-induced forcing, rather than phytoplankton host specificity (4). There is an association between a large abundance of bacteria and phytoplankton blooms after their peak and during the declining phase. Substrates released from degrading algae thereby support bacterial growth (6–8). However, bacteria often also play active roles in the shaping of plankton communities. Symbiotic, mutualistic, competitive, and predatory interactions can directly affect communities of primary producers (9), especially with algicidal bacteria, which have the potential to modulate entire phytoplankton communities. Algicidal bacteria can inhibit microalgal growth and use algal exudates as a resource or they can actively lyse the algae and utilize the released nutrients (10). Research on this class of bacteria is often motivated by potential applications in biotechnology, environmental engineering, and aquaculture. In particular, the possibility of being able to control harmful algal blooms by using algicidal bacteria that act against specific hosts is under discussion (11). As a consequence, knowledge has been accumulated mostly in bilateral alga-bacterium studies, and studies mostly exclude the dynamics of algicidal bacterial growth and the influence of the bacteria on the complex phytoplankton community in nature.

The mode of action of algicidal bacteria can be categorized in local interactions relying on direct contact of bacteria with their target algae or phycosphere interactions within the immediate vicinity of the cells. However, free-living bacteria can also become harmful to phytoplankton when they reach high cell concentrations. A recent review summarized the diversity of bacterial strategies and their ecological relevance in negatively affecting algal growth (10). The specificity of algicidal bacteria varies profoundly, from highly specialized bacteria that are associated with one host species to nearly universally active bacteria that affect most phytoplankton community members. The algicidal flavobacterium Kordia algicida, which is the subject of the current study, was isolated during a bloom of the diatom Skeletonema costatum (12). It is capable of lysing a broad range of algal species, including the diatoms Skeletonema costatum, Thalassiosira weissflogii, and Phaeodactylum tricornutum (13). The lysis is dependent on bacterial protease activity, regulated in a quorum-sensing-dependent manner (13). While most tested algae were lysed by K. algicida or its extracts, the diatom Chaetoceros didymus was resistant. Resistance was associated with upregulation of proteases from the algae, and these proteases are suspected to counteract the enzymes of the bacteria (14). In a second line of defense, oxylipins from the diatom can contribute to the resistance (15).

The specific resistance of Chaetoceros didymus prompted us to ask the question of how the bacteria mediate natural plankton populations with mixed assemblages of resistant and susceptible species. Considering the fact that microalgae compete for light and nutrients during bloom formation, removing one or more species from the assemblage might lead to dramatic shifts within the phytoplankton consortium.

In this study, we hypothesized that parts of the resistant phytoplankton community would benefit from the lysis of susceptible species. Here we tested whether and how this might lead to a population shift, thereby contributing to the understanding of the specificity and complexity of alga-bacterium interactions. We selected the especially well-characterized plankton community of Helgoland, which has been closely monitored for more than 50 years, and manipulated it with the algicidal bacterium K. algicida, which is globally distributed and lyses diatoms with high efficiency (3). Knowledge about cascading effects within microbial communities in the environment will help to explain natural patterns in plankton development.

## RESULTS

### Dominant phytoplankton members at Helgoland Roads.

**FIG 1:**
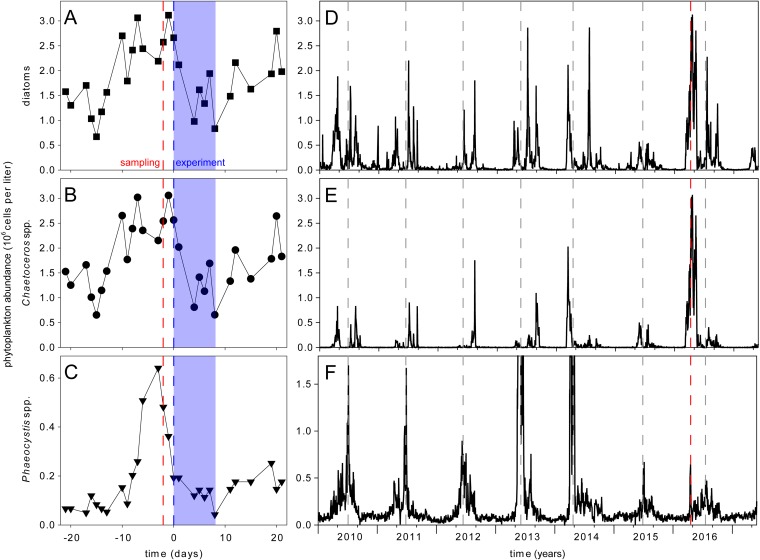
Phytoplankton abundance at Helgoland Roads. Cell counts of total diatoms (A and D), *Chaetoceros* spp. (B and E), and *Phaeocystis* spp. (C and F) are depicted. The day of sampling (red dashed lines), the day of bacterial inoculation (blue dashed lines), and the duration of the experiment (blue shaded areas) are highlighted. Annual events of high abundances of *Phaeocystis* spp. are marked with gray dashed lines. In 2013 and 2014, the abundance of *Phaeocystis* spp. reached 15.2 × 10^6^ and 5.7 × 10^6^ cells per liter, respectively (F).

Plankton community enclosures were set up during the time of a naturally occurring bloom of *Chaetoceros* spp. just after the peak of a bloom of *Phaeocystis* spp., using water from the site where samples for species enumeration were taken (Fig. 1). Diatoms at Helgoland Roads at the end of April 2016 were dominated by *Chaetoceros* spp. (Fig. 1A and B). *Chaetoceros* spp. in outside waters decreased slightly during the time of our experiment (Fig. 1B). Between sampling and inoculation of the enclosures, the bloom of *Phaeocystis* spp. outside declined further and then stayed comparatively stable over the course of the experiment (Fig. 1C). After the experiment, *Chaetoceros* spp. continued to grow in the outside waters until collapsing 1 month later (Fig. 1E). *Phaeocystis* spp. in the outside waters increased before peaking again approximately 3 months later (Fig. 1F).

### Phytoplankton community patterns in enclosures.

We monitored the phytoplankton community by light microscopy at a species or genus level. The survey included 12 diatoms, 7 dinoflagellates, 3 cryptophytes, 1 chlorophyte, 1 haptophyte, and 1 raphidophyte, thereby covering the most abundant species (Table 1).

**TABLE 1:** Identified phytoplankton species in enclosures

Category	Genus/species
Diatoms	*Cerataulina pelagica*
	*Chaetoceros socialis*
	*Cylindrotheca closterium*
	*Delphineis surirella*
	*Ditylum brightwellii*
	*Leptocylindrus danicus*
	*Leptocylindrus minimus*
	*Nitzschia* sp.
	*Podosira* sp.
	*Pseudo-nitzschia delicatissima*
	*Pseudo-nitzschia seriata*
	*Seminavis robusta*
Dinoflagellates	*Amphidinium* sp.
	*Gymnodinium* sp.
	*Mesoporos* sp.
	*Noctiluca* sp.
	*Polykrikos* sp.
	*Prorocentrum balticum*
	*Protoperidinium drevipes*
Cryptophytes	*Hemiselmis* sp.
	*Plagioselmis* sp.
	*Teleaulax* sp.
Raphidophytes	*Heterosigma niei*
Haptophytes	*Phaeocystis* sp.
Chlorophytes	*Pyramimonas* sp.

**FIG 2:**
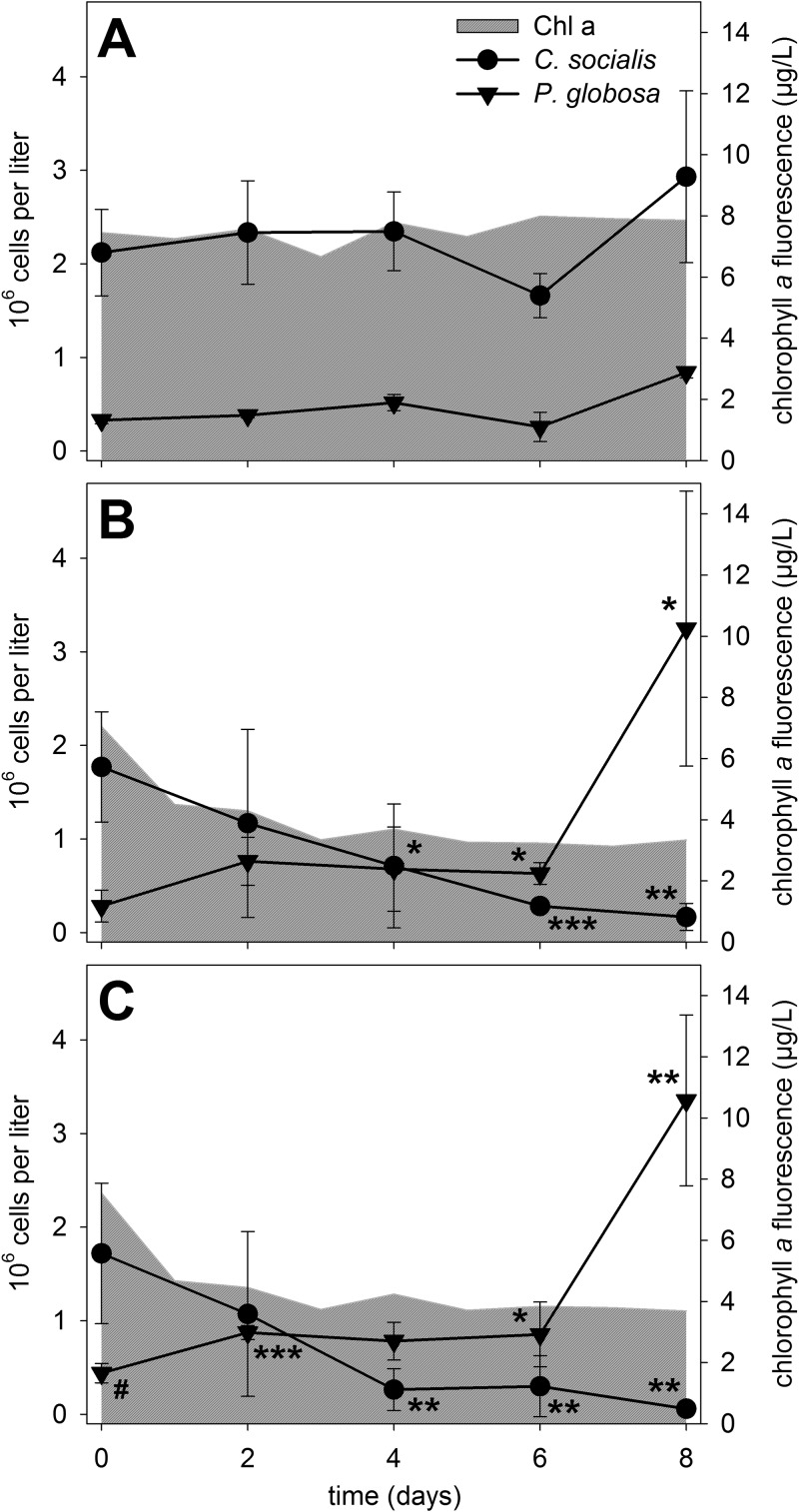
Phytoplankton development after bacterial infection. *Chaetoceros socialis* and *Phaeocystis* sp. cell counts (means ± standard deviations [SDs] of three biological replicates) and total chlorophyll *a* (Chl a) fluorescence (means of three biological replicates) are depicted for the control (A), the low infection scenario (B), and the high infection scenario (C). Significant differences in cell counts were tested via unpaired two-sided *t* tests, compared to the control treatment, for each time point and are indicated by asterisks (*, *P* < 0.05; **, *P* < 0.01; ***, *P* < 0.001) (see Table S4 in the supplemental material). The number sign marks a data point without normal distribution. Further statistical analyses of cell counts and chlorophyll *a* levels for the comparison between treatments (B and C) and for assessment of control stability (A) are given in Tables S1 to S4.

At the beginning of our experiment, the community was dominated by the diatom Chaetoceros socialis (∼2 × 10^6^ cells per liter) (Fig. 2) and the haptophyte *Phaeocystis* sp. (3 × 10^5^ to 4 × 10^5^ cells per liter) (Fig. 2). The species composition in the enclosures at the start of the experiment was thus in accordance with the cell abundances from the outside waters (Fig. 1). Microscopic evaluation of plankton samples before Lugol staining revealed that nearly all *Phaeocystis* sp. cells were found in colonies.

C. socialis abundance was stable in control enclosures throughout the experiment, as were *Phaeocystis* sp. concentrations. A significant increase (*P* value of <0.001, compared to day 0) was seen only on day 8 (Fig. 2A; also see Table S1 and Table S2 in the supplemental material). The stable cell counts of the two most dominant species were reflected in the uniform total chlorophyll *a* contents of the samples throughout the experiment. This stability was a prerequisite for evaluation of the effects of the phytoplankton assemblage manipulation by addition of K. algicida, which was previously isolated during a bloom of the diatom Skeletonema costatum (12). The effects of the bacteria on total chlorophyll *a* concentrations, on individual phytoplankton species, on the bacterial community, and on nutrient levels were monitored and revealed significant changes induced by the introduced bacteria.

Infecting the enclosures with K. algicida substantially changed the phytoplankton community composition over the following 8 days. In both infection scenarios, i.e., with high bacterial cell densities (final optical density at 550 nm [OD_550_] of 0.02) and low cell densities (final OD_550_ of 0.01), the total chlorophyll *a* contents decreased significantly (*P* values of <0.001), compared to the control, within 24 h (Fig. 2B and C; also see Table S3). In both infection scenarios, chlorophyll contents remained significantly lower (*P* values of <0.001) than the control levels throughout the experiment (except in the low infection scenario versus control on day 6, with data not normally distributed). From day 5 to day 7, there was significantly lower chlorophyll *a* fluorescence in the low infection scenario than in the high infection scenario.

In both infection scenarios, the abundance of C. socialis decreased to below 10% of the initial cell counts over the course of the experiment (Fig. 2B and C). C. socialis abundance was significantly lower than the control with both treatments from day 4 onward. There was no significant difference in C. socialis cell density between the two infection scenarios at any time point (Table S4).

*Phaeocystis* sp. cell counts doubled within 2 days after infection (significantly different from the control only for the high infection scenario), and then cell counts stayed constant until there was a drastic increase at day 8 to reach ∼10-fold greater concentrations, compared to day 0 (Fig. 2B and C). From day 6 onward, *Phaeocystis* sp. cell densities were significantly higher than the control levels in both infection scenarios, while there was no statistically significant difference between the two infection scenarios at any time point (Table S4). The increase in *Phaeocystis* sp. levels at day 8 was not reflected in total *in situ* chlorophyll *a* levels, which is in agreement with previous findings (16). The sum of all other phytoplankton species did not exceed 7 × 10^5^ cells per liter in control or infected enclosures at any time of sampling. Due to mainly episodic occurrences of these minor phytoplankton species, no obvious trend in cell counts that was dependent on the treatment was observed.

### Total bacterial abundance.

**FIG 3:**
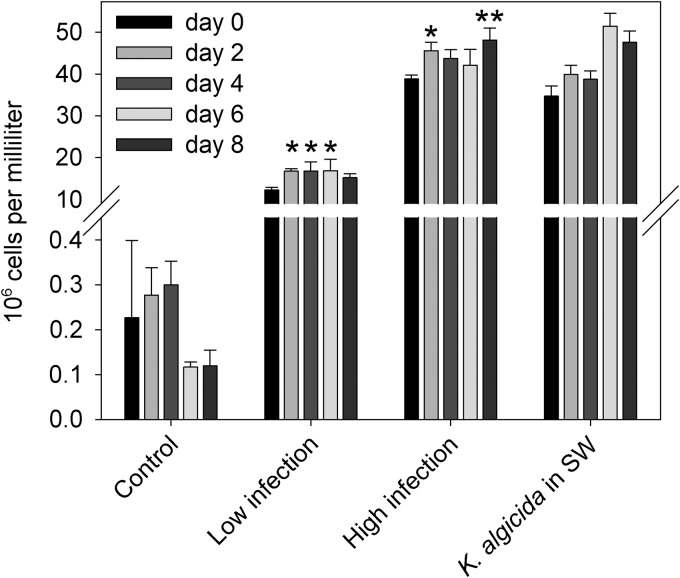
Bacterial abundance in plankton community enclosures. Total bacterial abundances were determined by flow cytometry. Cell counts are given for control enclosures, both infection scenarios, and treatment with a high K. algicida concentration in sterile filtered seawater (SW). Error bars denote SDs between biological triplicates except for K. algicida in seawater, which was technically replicated (means ± SDs of three replicates; no statistical test was applied for this treatment). Significant differences from one-way ANOVA, compared to day 0 for the respective treatment, are indicated (*, *P* < 0.05; **, *P* < 0.01) (see Table S5 in the supplemental material).

Bacterial abundance was monitored as a group parameter throughout the experiment. Total bacterial abundances in control enclosures remained constant throughout the 8 days of incubation (*P* = 0.075, analysis of variance [ANOVA]) (Table S5), compared to day 0 (Fig. 3). With low and high K. algicida inoculations, the bacterial densities increased 100- and 200-fold, respectively, compared to control levels (Fig. 3), thus rendering K. algicida the dominant bacterial species after infection. Bacterial densities in the high infection scenario were similar to those in the control in which K. algicida was added to sterile filtered seawater. Bacterial abundance in K. algicida-treated enclosures with the natural plankton community increased slightly but significantly during the experiment (Fig. 3; also see Table S5).

The viability of K. algicida was confirmed at the end of the experiment by plating of bacteria from high and low infection scenarios. In this nonquantitative survey, K. algicida colonies were clearly identifiable by their yellow pigmentation and colony morphology. No such colonies were found when a sample from the natural community was plated. The 16S rRNA sequencing of five randomly picked yellow colonies and five morphologically different colonies confirmed the identification. We found the majority of the colonies in the treatments to be K. algicida, confirming the survival of the bacteria throughout the experiment.

### Nutrients.

Levels of dissolved organic carbon (DOC) and inorganic nitrogen and phosphate in control enclosures containing the natural phytoplankton community did not change significantly over the course of the experiment (Table 2; also see Table S6 and Table S7). Elevated DOC contents were found in infected natural communities and controls in which K. algicida was introduced into sterile filtered seawater. DOC levels with these treatments decreased over the course of the experiment (day 4, *P* values of ≤0.01 for both; day 8, *P* values of ≤0.004 for both) (Table 2; also see Table S6). Initial concentrations of inorganic nutrients were higher in treatments with K. algicida inoculation than in the seawater control (Table 2). The inoculation with K. algicida in sterile filtered seawater resulted in elevated nitrite, ammonium, and phosphate concentrations, presumably due to carryover of nutrients associated with the bacterial cells. Lysis of the phytoplankton community contributed to a further substantial and significant increase in nutrients. Comparison of the infection scenario to K. algicida inoculation in sterile filtered seawater revealed that ∼40% of the additional phosphate could be attributed to nutrient release during infection. For ammonium, ∼20% of the surplus could be attributed to release during infection. Ammonium concentrations in infected plankton consortia were significantly increased at day 4 (*P* = 0.022) and day 8 (*P* = 0.011), compared to initial values (Table S8). Nitrate concentrations did not exceed the lower working limit of 7.14 µM in any treatment.

**TABLE 2:** Concentrations of nutrients

Sample and day	Level (mean ± SD)^*a*^			
	NO_2_^−^ (µM)	NH_4_^+^ (µM)	PO_4_^3−^ (µM)	DOC (mg liter^−1^)
Control (natural community)				
0	ND	ND	0.09 ± 0.03	2.65 ± 0.25
4	ND	ND	0.09 ± 0.01	2.17 ± 0.14
8	ND	ND	0.09 ± 0.01	2.05 ± 0.34
High infection				
0	0.51 ± 0.15	23.10 ± 4.18	1.32 ± 0.05	4.57 ± 0.06
4	0.42 ± 0.05	34.57 ± 5.18	1.55 ± 0.21	3.45 ± 0.62
8	0.36 ± 0.06^*b*^	38.91 ± 4.30	1.71 ± 0.07	2.94 ± 0.17
*K. algicida* in sterile filtered seawater				
0	0.33 ± 0.02^*b*^	18.71 ± 5.05^*b*^	0.84 ± 0.01	4.15 ± 0.03
4	0.33 ± 0.04^*b*^	30.25 ± 0.35^*b*^	0.89 ± 0.01	3.09 ± 0.04
8	0.32	29.00 ± 4.75^*b*^	1.09 ± 0.02	3.41 ± 0.05

a Values represent the means ± SDs of three biological replicates for the control and the high infection scenario except for nitrite at high infection on day 8 (two replicates). Concentrations of nutrients in *K. algicida* in sterile filtered seawater were obtained from technical replicates (means ± SDs of two or three measurements; one value at day 8 for nitrite). The lower limits of the working area were 0.29 µM for NO_2_^−^ and 3.57 µM for NH_4_^+^. ND, not detected. Nitrate levels were below 7.14 μM over the course of the experiment.

b Duplicates.

### Confirming laboratory experiments.

Following the field experiment, C. socialis was isolated from the control enclosures. The identity of C. socialis was confirmed by genetic analysis. According to a BLAST search using the D1-D3 large subunit (LSU) rDNA sequence (GenBank accession no. MH992142) as a query, the most similar sequence (GenBank accession no. JQ217339.1) (17), with 99% identity, originated from C. socialis. Subsequent laboratory experiments with the monoclonal culture supported the findings of the enclosure experiment. C. socialis was susceptible to K. algicida under conditions similar to those in the field and was lysed rapidly after inoculation with the bacterium (Fig. S1).

## DISCUSSION

Algicidal bacteria are important players in marine ecosystems. To date, however, they have mainly been studied in simplified bioassays with isolated species. Investigations thus focus on rather artificial interactions between two partners, without taking into account potential cascading effects that might occur in the natural surroundings (10). This is also the case for the flavobacterium K. algicida, which has been tested with regard to its activity on different isolated phytoplankton species. Those previous investigations revealed that, of the four species tested, only Chaetoceros didymus was resistant (13). The approach of community enclosures and manipulation presented in this study overcame the experimental limitations of few interaction partners in previous infection experiments. This enabled an evaluation of whether species-specific resistance is also observed in nature and how such resistant species respond to the lysis of surrounding cells. We could observe direct and cascading effects after infection of a natural *Chaetoceros* bloom with the algicidal bacterium. C. socialis cell counts decreased dramatically after inoculation with K. algicida, compared to the stable counts in the control. The treatment caused an increase in *Phaeocystis* sp. cell counts; this species obviously benefited from lysis of the competing dominant C. socialis. Interestingly, *Phaeocystis* sp. levels were already declining in the outside waters at the start of the experiment. Removal of the competing C. socialis by the algicidal bacteria apparently restored favorable conditions. The algicidal bacteria thus have the potential to shift the entire plankton population, since resistant species rapidly take over the liberated resources and benefit from the removal of competitors.

The long-term phytoplankton survey at Helgoland Roads conducted by the Biologische Anstalt Helgoland (18), with its daily screening, permitted us to time the experiment during a diatom bloom. *Chaetoceros* species often occur in the spring around March to May. The peak of the bloom in 2016 defined the beginning of our experiment. The bloom in 2016 was the most intensive one recorded within the monitoring period (from 2010 to 2017) (Fig. 1). The blooming species was confirmed to be C. socialis and was the most abundant algal species, in terms of cell numbers, in the sea and in the enclosure experiments at the start of our experiment. Total diatom cell counts and *Chaetoceros* cell counts were virtually superimposable during the course of the experiment, indicating that the diatom community was consistently dominated by this species (Fig. 1A and B). The *Chaetoceros* genus is particularly important at Helgoland Roads and the habitat hosts different *Chaetoceros* species, with each showing an individual growth pattern throughout the year (3). The second dominant species was *Phaeocystis* sp., which was already in the declining phase of a bloom that peaked before the start of our experiment (Fig. 1). To study specifically the effect of the introduced algicidal bacterium in our experiment, we limited grazers by filtering the water through 100-µm filters, to remove the majority of herbivores. The limited effect of grazing was confirmed by the stable cell counts in the control enclosures (Fig. 2A). We also added the bacteria at a high optical density, to ensure that immediate bloom termination was achieved and to exclude overlaying effects of competing bacteria. The success of this strategy was documented by the fact that the two bacterial treatments (high and low infection scenarios) caused similar effects (Fig. 2).

The susceptibility of C. socialis was initially unexpected, since the only tested diatom of the same genus, C. didymus, was resistant to K. algicida in a laboratory screening (13). However, such variability in algal host susceptibility toward algicidal bacteria, even within isolates from the same species, has been already documented in the literature (19). Apart from K. algicida, other bacteria are known to affect *Chaetoceros* negatively; thus, this genus has no outstanding resistance traits (20–22). In contrast, *Phaeocystis* sp. was not lysed by the bacterium and cell counts increased immediately on day 1 after lysis of the diatoms. The observed host specificity is in agreement with laboratory experiments on the algicidal bacterium *Brevibacterium* sp., which is active against a broad selection of microalgae, including a *Chaetoceros* sp., but does not affect the *Phaeocystis* sp. (23). *Phaeocystis* resistance has been connected to the ability to form large colonies. In colonies, cells produce transparent exopolymer particles (TEPs) that protect the colony from infections (24, 25). TEPs have been directly connected to physical protection by scavenging bacteria and viruses (26, 27). The algicidal agent excreted by K. algicida is a protease (13), and the saccharide-rich exopolymeric matrix might prevent the protease from reaching cellular structures by limiting diffusion (28).

The kinetics of the C. socialis decline and the rise of *Phaeocystis* were strikingly similar for the two infected treatments, and we conclude that the inoculation density was already sufficiently high in the low inoculation scenario to trigger a maximum effect. *Phaeocystis* bloomed twice in 2016, once in parallel with C. socialis and once ∼3 months after the experiment, when the diatom spring bloom was over. The algicidal bacterium thereby accelerated the natural succession of *Phaeocystis*, inducing the second bloom already a few days after the decline of the diatoms that was triggered by the bacterium. Such community manipulations by algicidal bacteria might have to be generally considered in Helgoland and northern waters, and future studies should include the monitoring of algicidal bacteria during bloom decline. In the period from 2010 to 2016, *Phaeocystis* often followed the diatom blooms in Helgoland (with the exception of 2012 and 2016, when they cooccurred) (Fig. 1) (3, 4, 29). This seasonal succession was confirmed at seven long-term monitoring stations along the North Sea coast, where a diatom-dominated bloom was often followed by a *Phaeocystis* bloom and then by mixed blooms in summer (29). Such patterns also hold true for other phytoplankton field studies apart from North Sea waters (30–33).

Compared to diatoms, *Phaeocystis* species have greater light requirements and therefore should be outcompeted during the low-light conditions of a dense spring bloom of other species (34). The addition of K. algicida removes the most dominant competitor of the *Phaeocystis* sp. from our enclosure experiments and we assume allows the *Phaeocystis* sp. to thrive due to improved light availability. This effect would be relevant in our experiments, where we simulated low-light conditions in the turbid coastal environment at Helgoland Roads (35). This idea is supported by the previous evaluation of 50 years of high-resolution data, demonstrating that light is the main driver for phytoplankton dynamics at Helgoland Roads (3).

A second factor supporting the *Phaeocystis* bloom can be the availability of nutrients. We detected higher nutrient concentrations after bacterial infections. Portions of the nutrients stem from carryover during infection. The additional increases of nutrients and DOC in the infected enclosures indicate contributions of cellular nutrients released during the rapid lysis of C. socialis (Table 1). Already within the first day, a 38% decrease in total chlorophyll *a* fluorescence was observed in the high infection scenario. The associated cell lysis liberated substantial amounts of intracellularly stored nutrients and DOC. Diatoms are known to efficiently store nutrients such as nitrate, which can be liberated upon lysis (36). Adding to the complexity of the system, however, even ammonium has been discussed recently as an algicide (37). Excreted bacterial proteases that mediate phytoplankton lysis can also contribute to higher initial DOC concentrations (13). DOC levels were lower at the end of the experiment (64% of initial levels) in the treatments in which the algal community was exposed to K. algicida, compared to the control in which K. algicida was added to sterile filtered seawater (82% of initial levels). The observed bacterial growth and/or the blooming *Phaeocystis* likely contributed to the consumption. A long-standing hypothesis claims that growth of *Phaeocystis* may be dependent on dissolved organic matter from decaying diatom blooms (38, 39). Haptophytes in general have been linked to a mixotrophic lifestyle (40, 41). Our data are in accordance with this hypothesis, which would explain the onset of *Phaeocystis* growth right after the decline of the diatoms. Signaling metabolites such as quorum-sensing molecules or allelopathic chemicals also might contribute to the observed succession, but these multiple factors cannot be fully untangled with the enclosure experiments.

In conclusion, we document that algicidal bacteria can shift natural plankton populations and accelerate plankton succession. In Helgoland waters, the introduction of K. algicida led to an accelerated bloom decline of the dominant diatom C. socialis and the earlier onset of *Phaeocystis* sp. Resistance to the bacteria thus provides a competitive advantage in the multispecies communities of the plankton.

## MATERIALS AND METHODS

### Experimental design.

To start the enclosure experiments, a natural seawater community was taken from subsurface water during the phytoplankton spring bloom period at the research site Kabeltonne at Helgoland Roads in the German North Sea (54˚11.3′N, 7˚54.0′E) on 19 April 2016. The collection procedure and sampling site were the same as described for routine sampling during the long-term plankton monitoring at the Biologische Anstalt Helgoland (42, 43). Polycarbonate vessels (25 liters; Nalgene, Rochester, NY, USA) were acid washed (10% hydrochloric acid), rinsed with ultrapure water, and autoclaved. Before filling, the vessels were additionally washed with natural seawater, and natural plankton samples were filtered (100-µm mesh size) to exclude macro-zooplankton. Nine culturing vessels were set up, each containing 20 liters of the resulting natural community. Additionally, two control vessels with sterile filtered seawater (20 liters each) were prepared by further filtration through 1.2-µm filters (GF-C; Whatman, Kent, UK), followed by filtration through 0.2-µm filters (Filtropur S; Sarstedt, Nümbrecht, Germany) under reduced pressure. In sum, 11 vessels containing either the natural community or sterile filtered seawater were set up in a climate chamber (7.6°C, light/dark cycle of 15 h/9 h, and light intensity of 3 µmol × m^−2^ × s^−1^). Treatments were acclimated for 2 days until the experiment was started by inoculation with K. algicida. K. algicida was plated 4 days in advance on petri dishes with solid marine broth agar and was cultured at room temperature. Colonies were washed off with sterile filtered seawater on the day of inoculation. This procedure was applied for approximately 200 plates (10-cm diameter) with bacterial lawns, resulting in a dense bacterial solution that was subsequently diluted (1:20 [vol/vol]) to reach a final OD_550_ of 0.43. This bacterial suspension was added to bottles filled with the natural community to a final OD_550_ of 0.01 (23 ml; low treatment [*n* = 3]) or 0.02 (46 ml; high treatment [*n* = 3]). A vessel containing sterile filtered seawater was inoculated with bacterial solution to a final OD_550_ of 0.02 (K. algicida control [*n* = 1]). Three vessels containing the natural community and one containing sterile filtered seawater served as controls and were not treated with K. algicida. Prior to sampling, all vessels were briefly and vigorously shaken (horizontally) manually every day, and their position in the climate chamber was subsequently exchanged. Total chlorophyll *a* levels, levels of individual phytoplankton species, total bacterial abundance, the presence of K. algicida, inorganic nitrogen and phosphate levels, and dissolved organic matter (DOC) levels were followed over the course of the experiment (8 days). Sampling was performed every second day except for chlorophyll *a* fluorescence, which was determined daily.

### Community responses.

As a proxy for phytoplankton abundance, *in situ* chlorophyll *a* fluorescence was monitored daily. For chlorophyll *a* measurements, 30 ml from each sample was directly quantified with a BBE algae laboratory analyzer (Moldaenke, Schwentinental, Germany). Every second day, cell identification and enumeration were performed using light microscopy. Phytoplankton cell identification and enumeration were carried out at the species or genus level using sedimentation chambers on an inverted microscope (IM35 [Carl Zeiss, Jena, Germany] or DM IL LED [Leica Microsystems GmbH, Wetzlar, Germany]), at ×400 magnification. Sedimentation chambers of 10 ml and 25 ml were topped up with samples after fixation with Lugol’s solution and were left undisturbed to settle for 12 h and 24 h, respectively. The phytoplankton content of each sample was identified with the guide of identification keys (44–47).

For the analysis of total bacterial abundance, subsamples (1 ml) were gently filtered manually through membranes with a pore size of 5 µm (13-mm Nuclepore Track-Etch membranes; Whatman), placed in a syringe filter holder (13-mm Swinnex filter holder; Merck, Darmstadt, Germany). After fixation with 25% glutaraldehyde to a final concentration of 2%, the samples were stored at −20°C. Prior to the analysis by flow cytometry (BD Accuri C6; BD Bioscience, Heidelberg, Germany), samples were thawed at 37°C for 10 min and diluted in TE buffer (10 mM Tris-HCl [pH 7], 1 mM EDTA [pH 8]) either 1:10 when no K. algicida was added, 1:20 when K. algicida was added at a low concentration, or 1:50 when K. algicida was added at a high concentration. All samples were stained with Sybr Gold (at a 1:10,000 dilution of the stock solution supplied by the manufacturer; Thermo Fisher Scientific, San Jose, CA, USA) for 10 min at 80°C. Each sample (50 µl) was measured at a flow rate of 35 µl min^−1^, leading to rates of 500 to 2,000 events × s^−1^.

K. algicida was additionally monitored by plating 50 µl of one unfiltered representative sample for each of the five treatment groups (natural community, K. algicida high concentration, K. algicida low concentration, K. algicida high concentration in sterile filtered seawater, and sterile filtered seawater) on marine broth agar at three dilutions (1:1,000, 1:10,000, and 1:100,000 in sterile filtered seawater). Plates were incubated for up to 3 days at 30°C, checked for the presence of K. algicida colonies, and finally stored at 4°C. K. algicida colonies were identified by their yellow color and their colony morphology. Colonies are round (configuration), entire (margin), and slightly convex (elevation) (12). Genetic identification of random representative colonies was performed by sequencing 16S rRNA. Genomic DNA was isolated from single colonies using a NucleoSpin Tissue XS kit (Macherey-Nagel GmbH & Co. KG, Düren, Germany), according to the manufacturer’s instruction with the following exceptions: prelysis of samples was performed overnight, and optional removal of residual ethanol after elution was performed. For 16S rRNA amplification, the universal primers 27f (AGA GTT TGA TCA TGG CTC A) and 1392r (ACG GGC GGT GTG TGT AC) were ordered from biomers.net GmbH (Ulm, Germany). The PCR mixture contained One*Taq* standard reaction buffer, 200 µM deoxynucleoside triphosphates (dNTPs), 0.2 µM each primer, and 1.25 U One*Taq* DNA polymerase (New England Biolabs, Frankfurt, Germany). The temperature program was as follows: initial denaturation at 95°C for 2 min; 30 cycles of denaturation at 95°C for 30 s, annealing at 58°C for 30 s, and elongation at 68°C for 90 s; and finally elongation at 68°C for 5 min. The PCR product was purified from a HDGreen-stained (Intas Science Imaging Instruments GmbH, Göttingen, Germany) 1% agarose gel using a GenElute gel extraction kit (Sigma-Aldrich, Munich, Germany), according to the manufacturer’s instructions. Sanger sequencing was performed by GATC Biotech AG (Konstanz, Germany) using primer 27f. Sequences were manually edited in BioEdit (48), and the final sequence was subjected to a BLAST search (BLASTn and Megablast) against the nucleotide collection (nonredundant/nucleotide) (49) between 24 March 2017 and 21 April 2017.

### DOC and inorganic nutrients.

Subsamples (40 ml) for the analysis of DOC were sterile filtered (Filtropur S) after sampling and stored at −20°C. They were thawed before analysis with an ion chromatography system on an Elementar vario TOC cube (working range, 0.1 to 50 mg × liter^−1^; Elementar Analysensysteme, Langenselbold, Germany). Sample measurements were performed in duplicate using a sample volume of 10 ml. Subsamples (40 ml) for the analysis of inorganic nutrients (nitrate, nitrite, ammonium, and phosphate) were directly stored at −20°C after sampling. They were thawed and filtered through 0.45-µm filters (Chromafil Xtra PET; Macherey-Nagel GmbH & Co. KG) before analysis using flow injection on a Quikchem QC 8500S2 instrument (Lachat Instruments, Loveland, CO).

### Genetic identification of *Chaetoceros socialis*.

Chaetoceros socialis was isolated from the natural community after the end of the experiment. The culture was maintained in our culture collection according to published procedures (50).

Cells of Chaetoceros socialis were concentrated by centrifugation (850 × *g* at 4°C for 20 min), and the cell pellets were frozen (−80°C) and freeze-dried. Dried cell pellets were lysed with zirconium beads (0.9 mm) for 1 min at 30 Hz in a TissueLyser II (Qiagen, Venlo, Netherlands) that had been precooled to −80°C. Genomic DNA was isolated using the Isolate II plant DNA kit (Bioline GmbH, Luckenwalde, Germany) with lysis buffer PA1 without RNase. D1-D3 LSU rDNA was amplified based on an established protocol (51). The primers D1R-F (ACC CGC TGA ATT TAA GCA TA) (52) and D3B-R (TCG GAG GGA ACC AGC TAC TA) (51), obtained from biomers.net, were used. The PCR mixture contained One*Taq* standard reaction buffer, 200 µM dNTPs, 1 µM each primer, 20 mM tetramethylammonium chloride, and 1.25 U One*Taq* DNA polymerase (New England Biolabs). The temperature program was as follows: initial denaturation at 94°C for 2 min; 30 cycles of denaturation at 94°C for 30 s, annealing at 60°C for 30 s, and elongation at 68°C for 60 s; and finally elongation at 68°C for 5 min. The PCR product was purified from a HDGreen-stained (Intas Science Imaging Instruments) 1% agarose gel using the GenElute gel extraction kit (Sigma-Aldrich), according to the manufacturer’s instructions. Sanger sequencing was performed by GATC Biotech AG using the same primers as for PCR. Sequences were aligned using Clustal W (53) and were manually edited in BioEdit (48). The final sequence was subjected to a BLAST search (BLASTn and Megablast) against the nucleotide collection (nonredundant/nucleotide) (49) on 1 December 2016.

### Field data.

Total diatom abundance and cell counts for *Chaetoceros* spp. and *Phaeocystis* spp. in Fig. 1 were obtained as described previously (3). Data are deposited and updated regularly on the Pangea platform (18).

### Statistics.

Statistical analysis was conducted using SigmaPlot software (version 11 or higher; Systat Software Inc., London, UK). An unpaired two-sided *t* test was used to analyze the significance of differences in enclosure chlorophyll *a* levels and cell counts in infection scenarios, compared to the uninfected control, for each time point and species individually. Normal distribution (Shapiro-Wilk test) and equal variance of the data were tested in advance. If the normality test failed, then a Mann-Whitney rank sum test was performed instead. A one-way ANOVA was carried out for the analysis of control stability and for the comparisons of total bacterial abundances and DOC and ammonium concentrations over the course of the experiment; it was followed by either a Bonferroni or Holm-Sidak *post hoc* test when there were significant differences within the data set. Phosphate concentrations were tested using one-way ANOVA on ranks. Levels of significance are indicated, and *P* values of >0.05 were considered not significant. Data points in the figures without normal distribution are indicated.

### Accession number(s).

Sequence data were deposited in GenBank and are available under accession no. MH992142.

## Supplementary Material

Supplemental file 1
