# Supplementary material for: The Algicidal Bacterium Kordia algicida Shapes a Natural Plankton Community
Source: Appl Environ Microbiol. 2019 Mar 22;85(7):e02779-18. doi: 10.1128/AEM.02779-18 (PMC6585488; doi:10.1128/AEM.02779-18)
Supplement: Supplemental file 1 [file AEM.02779-18-s0001.pdf]

Supplementary Information

The algicidal bacterium *Kordia algicida* shapes a natural plankton community

Arite Bigalke[1]*, Nils Meyer[1]*, Lydia Papanikolopoulou[1], Karen Helen Wiltshire[2,3], Georg Pohnert[1,4#]

* contributed equally

[1]Institute for Inorganic and Analytical Chemistry, Bioorganic Analytics, Friedrich Schiller University Jena, Lessingstrasse 8, D-07743 Jena, Germany

[2]Alfred-Wegener-Institut, Helmholtz-Zentrum für Polar- und Meeresforschung, Biologische Anstalt Helgoland, P.O. Box 180, 27483, Germany

[3]Alfred-Wegener-Institut, Helmholtz-Zentrum für Polar- und Meeresforschung, Biologische Anstalt Helgoland, Wadden Sea station, 25992, List, Germany

[4]Max Planck Institute for Chemical Ecology, Hans Knöll Str. 8, D-07745 Jena, Germany

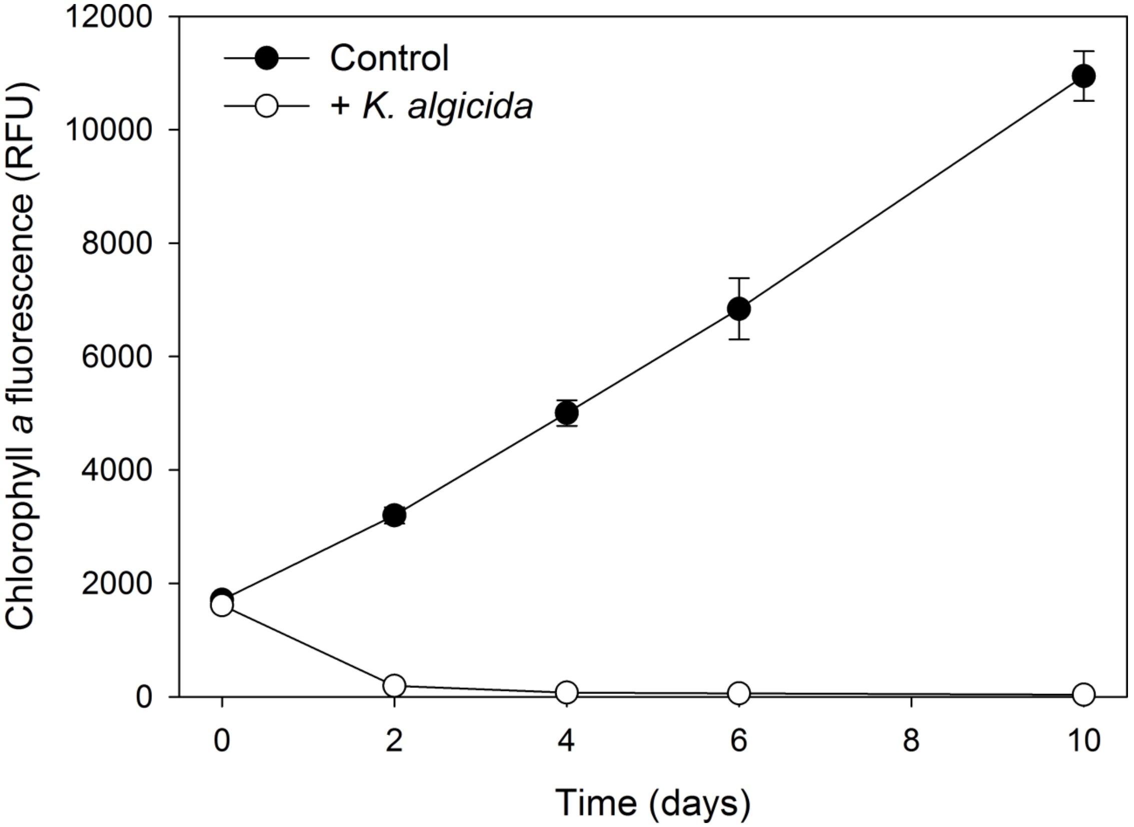

Fig. S1 *Chaetoceros socialis* mono culture in laboratory experiment. Cultures of *C. socialis* (40 ml, n=3) are grown in the presence ("+ *K. algicida*", final OD_{550} 0.01) or absence ("Control") of *Kordia algicida*.

Table S1: Statistics for stability of chlorophyll *a* fluorescence in control treatments. Given are p-values from Bonferroni *post-hoc* test after one-way ANOVA (P = 0.018).

comparison			p-value
day 0	vs.	day 1	1.000
day 0	vs.	day 2	1.000
day 0	vs.	day 3	0.219
day 0	vs.	day 4	1.000
day 0	vs.	day 5	1.000
day 0	vs.	day 6	0.950
day 0	vs.	day 7	1.000
day 0	vs.	day 8	1.000

Table S2: Statistics for stability of phytoplankton cell counts in control treatment. One-way ANOVA for *Phaeocystis* sp. cell counts displayed significant differences within the dataset ($P < 0.001$). *Chaetoceros socialis* cell counts were not significantly different in one-way ANOVA ($P = 0.176$). Given are p-values from Bonferroni *post-hoc* test of *Phaeocystis* sp.

comparison			p-value
day 0	vs.	day 2	1.000
day 0	vs.	day 4	0.070
day 0	vs.	day 6	1.000
day 0	vs.	day 8	< 0.001

Table S3: Statistical analysis of differences in chlorophyll *a* fluorescence between treatments. Given are two-tailed p-values from an unpaired t-test.

constant	Comparison			p-value
day 0	control	vs.	low *Kordia*	0.129
	control	vs.	high *Kordia*	0.792
	low *Kordia*	vs.	high *Kordia*	0.183
day 1	control	vs.	low *Kordia*	< 0.001
	control	vs.	high *Kordia*	< 0.001
	low *Kordia*	vs.	high *Kordia*	0.315
day 2	control	vs.	low *Kordia*	< 0.001
	control	vs.	high *Kordia*	< 0.001
	low *Kordia*	vs.	high *Kordia*	0.600
day 3	control	vs.	low *Kordia*	0.003
	control	vs.	high *Kordia*	0.003
	low *Kordia*	vs.	high *Kordia*	0.270
day 4	control	vs.	low *Kordia*	< 0.001
	control	vs.	high *Kordia*	< 0.001
	low *Kordia*	vs.	high *Kordia*	0.081
day 5	control	vs.	low *Kordia*	< 0.001
	control	vs.	high *Kordia*	< 0.001
	low *Kordia*	vs.	high *Kordia*	0.047
day 6	control	vs.	low *Kordia*	no normal distribution
	control	vs.	high *Kordia*	< 0.001
	low *Kordia*	vs.	high *Kordia*	0.008
day 7	control	vs.	low *Kordia*	< 0.001
	control	vs.	high *Kordia*	< 0.001
	low *Kordia*	vs.	high *Kordia*	0.007
day 8	control	vs.	low *Kordia*	< 0.001
	control	vs.	high *Kordia*	< 0.001
	low *Kordia*	vs.	high *Kordia*	0.074

Table S4: Statistical analysis of differences in phytoplankton cell counts (*Chaetoceros socialis* and *Phaeocystis* sp.) between treatments. Given are two-tailed p-values from an unpaired t-test.

species	constant	comparison			p-value
C. socialis	day 0	control	vs.	low *Kordia*	0.463
		control	vs.	high *Kordia*	0.474
		low *Kordia*	vs.	high *Kordia*	0.929
	day 2	control	vs.	low *Kordia*	0.153
		control	vs.	high *Kordia*	0.103
		low *Kordia*	vs.	high *Kordia*	0.907
	day 4	control	vs.	low *Kordia*	0.022
		control	vs.	high *Kordia*	0.002
		low *Kordia*	vs.	high *Kordia*	0.329
	day 6	control	vs.	low *Kordia*	<0.001
		control	vs.	high *Kordia*	0.005
		low *Kordia*	vs.	high *Kordia*	0.936
	day 8	control	vs.	low *Kordia*	0.007
		control	vs.	high *Kordia*	0.006
		low *Kordia*	vs.	high *Kordia*	0.261
P. globosa	day 0	control	vs.	low *Kordia*	0.697
		control	vs.	high *Kordia*	no normal distribution
		low *Kordia*	vs.	high *Kordia*	0.240
	day 2	control	vs.	low *Kordia*	0.064
		control	vs.	high *Kordia*	<0.001
		low *Kordia*	vs.	high *Kordia*	0.504
	day 4	control	vs.	low *Kordia*	0.577
		control	vs.	high *Kordia*	0.106
		low *Kordia*	vs.	high *Kordia*	0.735
	day 6	control	vs.	low *Kordia*	0.016
		control	vs.	high *Kordia*	0.048
		low *Kordia*	vs.	high *Kordia*	0.354
	day 8	control	vs.	low *Kordia*	0.047
		control	vs.	high *Kordia*	0.009
		low *Kordia*	vs.	high *Kordia*	0.920

Table S5 Statistical analysis of total bacterial abundances. One-way ANOVA within low *Kordia* treatment ($P = 0.033$) and high *Kordia* treatment ($P = 0.012$) over time displayed significant differences. In control mesocosms bacterial abundance was not significantly different between time points in one-way ANOVA ($P = 0.075$). Given are p-values from Bonferroni *post-hoc* test.

treatment	comparison			p-value
low *Kordia*	day 0	vs.	day 2	0.036
	day 0	vs.	day 4	0.034
	day 0	vs.	day 6	0.032
	day 0	vs.	day 8	0.247
high *Kordia*	day 0	vs.	day 2	0.037
	day 0	vs.	day 4	0.163
	day 0	vs.	day 6	0.621
	day 0	vs.	day 8	0.005

Table S6 Statistical analysis of DOC concentrations. One-way ANOVA within high *Kordia* treatment ($P = 0.005$) and *Kordia* in sterile-filtered seawater ($P < 0.001$) over time displayed significant differences. In control mesocosms DOC concentration was not significantly different between time points in one-way ANOVA ($P = 0.064$). Given are p-values from Holm-Sidak *post-hoc* test.

treatment	comparison			p-value
high *Kordia*	day 0	vs.	day 4	0.010
	day 0	vs.	day 8	0.004
Kordia in sterile-filtered seawater	day 0	vs.	day 4	< 0.001
	day 0	vs.	day 8	< 0.001

Table S7 Statistical analysis of phosphate concentrations. ANOVA on Ranks within *Kordia* in sterile-filtered seawater over time displayed significant differences (P = 0.025). Within control mesocosms (P = 0.929) and high *Kordia* treatments (P = 0.086) phosphate concentration was not significantly different between time points in ANOVA on Ranks. Given are p-values from Tukey *post-hoc* test.

treatment	comparison			p-value
Kordia in sterile-	day 0	vs.	day 4	0.822
filtered seawater	day 0	vs.	day 8	0.207

Table S8 Statistical analysis of ammonium concentrations. One-way ANOVA within high *Kordia* treatment over time displayed significant differences (P = 0.014). Given are p-values from Holm-Sidak *post-hoc* test.

treatment	comparison			p-value
high *Kordia*	day 0	vs.	day 4	0.022
	day 0	vs.	day 8	0.011
